# Knockdown of P4HA1 inhibits neovascularization via targeting glioma stem cell-endothelial cell transdifferentiation and disrupting vascular basement membrane

**DOI:** 10.18632/oncotarget.16270

**Published:** 2017-03-16

**Authors:** Yiqiang Zhou, Guishan Jin, Ruifang Mi, Junwen Zhang, Jin Zhang, Hengzhou Xu, Sen Cheng, Yunsheng Zhang, Wenjie Song, Fusheng Liu

**Affiliations:** ^1^ Brain Tumor Research Center, Beijing Neurosurgical Institute, Beijing 100050, P.R. China; ^2^ Department of Neurosurgery, Beijing Tiantan Hospital Affiliated to Capital Medical University, Beijing 100050, P.R. China; ^3^ Beijing Laboratory of Biomedical Materials, Beijing 100050, P.R. China

**Keywords:** glioma stem cells, neovascularization, transdifferentiation, P4HA1

## Abstract

Emerging evidence has demonstrated transdifferentiation process of glioma stem cells (GSCs) into endothelial cells (ECs) in glioma neovascularization. Herein, we focused on screening for genes that were differentially expressed in the transdifferentiation process using microarray analysis. Bioinformatics analysis revealed differential expression of the prolyl 4-hydroxylase subunit alpha-1 (P4HA1) gene. We determined that P4HA1 expression was correlated with histological grade, the level of Ki67 and microvessel density (MVD) in human glioma specimens. Knockdown of P4HA1 inhibited the proliferation, migration and tube formation of GSCs *in vitro*. *In vivo* studies revealed that the downregulation of P4HA1 inhibited intracranial tumor growth, prolonged the overall survival time of xenograft mice and suppressed the neovascularization in brain tumors. Moreover, P4HA1 regulates the expression of vascular endothelial growth factor A (VEGF-A), especially an anti-angiogenic isoform-VEGF165b. Additionally, knockdown of P4HA1 inhibited the synthesis of collagen IV, and hence disrupted the structures of vascular basement membranes (BMs) in gliomas. Our study indicates that P4HA1 plays a pivotal role in the process of GSC-EC transdifferentiation and the structural formation of vascular BMs.

## INTRODUCTION

Glioblastomas (GBMs) are the most aggressive brain tumors and account for 70% of the malignant primary brain tumors in adults [1]. Despite optimal treatment, the median survival of newly diagnosed GBM patients is less than 15 months [2]. As GBMs are highly vascularized tumors, anti-angiogenic therapies are of great interest [3]. However, few anti-angiogenic therapies have shown significant efficacy in clinical studies. Therefore, additional neovascular mechanisms and related genes need to be explored.

There are currently at least five known mechanisms of tumor neovascularization in GBMs: sprouting angiogenesis, vasculogenesis, vascular co-option, vascular mimicry (VM) and transdifferentiation of glioblastoma stem-like cells (GSCs) or GBM cells into endothelial cells (ECs) [4, 5]. The former three processes have been extensively described and are well recognized, and the latter two mechanisms are only beginning to be explored. The VM structure is a vessel-like network independent of ECs that is surrounded by tumor cells [6]. Both Ricci-Vitiani *et al*. [7] and Wang *et al*. [8] demonstrated that GSCs could transdifferentiate into ECs, and Ricci-Vitiani *et al*. postulated that VM represents an incomplete transdifferentiation of cancer stem cells into an endothelial phenotype. In addition, accumulating evidence indicates that hypoxia and hypoxia-inducible factors (HIFs) are implicated in all five processes of neovascularization [9–15]. Under hypoxia, GSCs or glioblastoma cells could be induced to express an endothelial phenotype [8–10]. Therefore, the transdifferentiation mechanism may be suspected to be involved in the failure of anti-angiogenic therapy for glioma.

In the current study, we aimed to identify the genes involved in transdifferentiation process. Microarray assays were performed to compare the gene expression profiles of GSCs and ECs (transdifferentiated from GSCs under hypoxia) to uncover candidate genes with significantly different expression levels. We found that the prolyl 4-hydroxylase subunit alpha-1 (P4HA1) was overexpressed in high-grade gliomas, and that P4HA1 expression was correlated with tumor microvessel density (MVD). Knockdown of P4HA1 could inhibit tumor growth and neovascularization *in vitro* and *in vivo*. Furthermore, we revealed that knockdown of P4HA1 could upregulate an anti-angiogenic factor, vascular endothelial growth factor 165b (VEGF_165_b), decrease the levels of collagen IV and disrupt the integrity of vascular basement membranes (BMs). Our study reveals a pivotal gene in glioma neovascularization mediated by GSC-EC transdifferentiation that influences the expression of VEGF_165_b and the integrity of vascular BMs. The P4HA1 gene may be a potential target for anti-angiogenic therapy in GBMs.

## RESULTS

### GSCs transdifferentiate into ECs *in vitro* and *in vivo*

**Figure 1:**
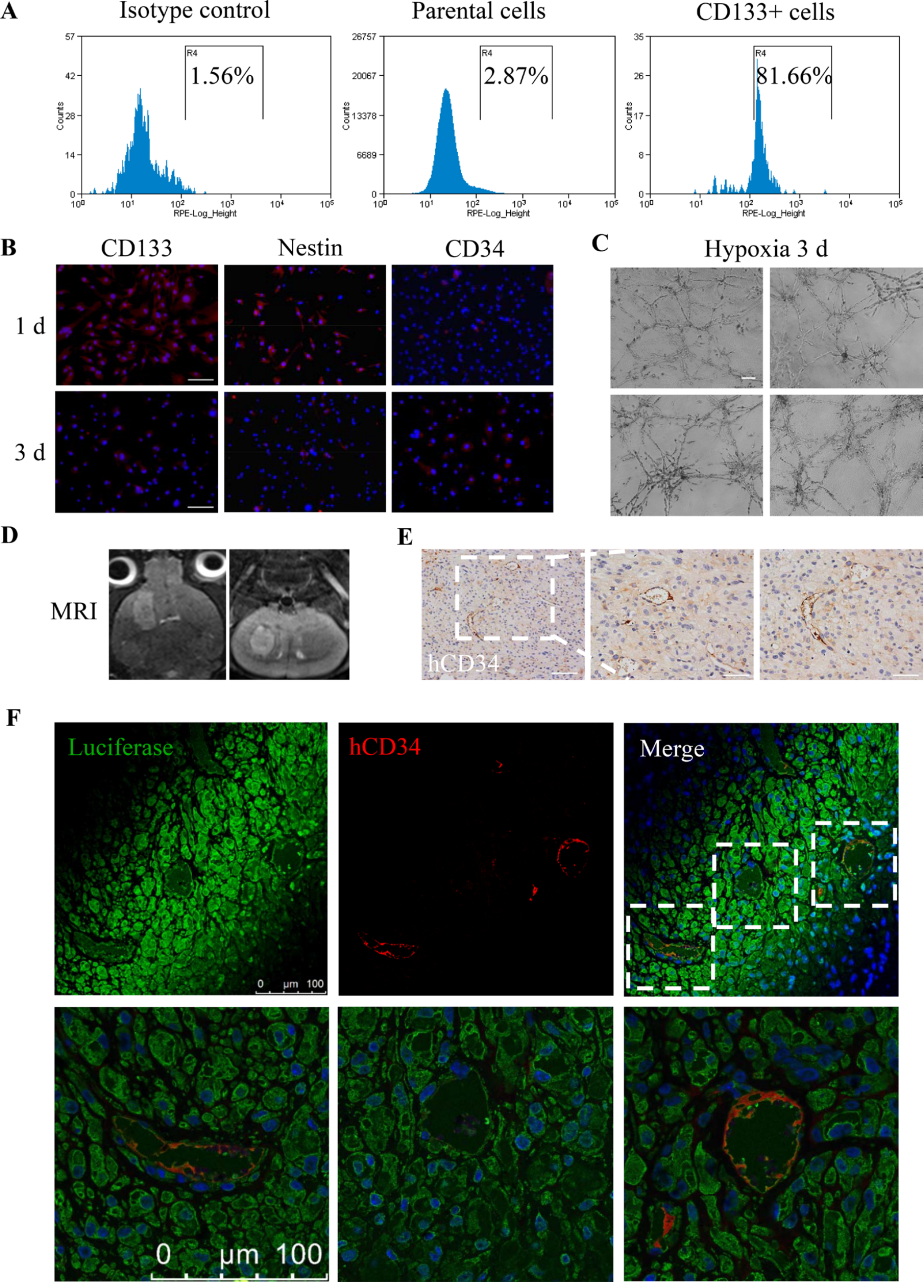
Transdifferentiation of GSCs into ECs *in vitro* and *in vivo* (**A**) CD133+ GSCs accounted for 1.31% of the parental U87MG cells, and sorting efficiency was 81.66%. (**B**) After incubation under 1% O_2_ for 3 days, the expression levels of CD133 and Nestin decreased, while the expression of CD34 increased. Scale bar = 50 μm. (**C**) CD133+ GSCs formed tube-like structures on Matrigel after treatment with 1% O_2_ for 3 days. Scale bar = 200 μm. (**D**) T2W images of a 28 day mouse intracranial tumor in coronal (left) and axial (right) views. (**E**) IHC analysis of hCD34 in intracranial tumor sections. Scale bar = 100 μm (left), 50 μm (right). (**F**) Co-expression of hCD34 and luciferase in the above tumor section detected by confocal microscopy. Scale bar = 100 μm.

To obtain sufficient GSCs, we sorted 1.31% CD133+ GSCs from parental U87MG cells via fluorescence-activated cell sorting (FACS) (Figure 1A) and incubated the CD133+ GSCs in serum-free medium. After the CD133+ GSCs were induced under hypoxia for 3 days, immunofluorescence (IF) was analyzed to detect the EC marker CD34 on day 1 and 3. We found that the levels of CD34 increased and the expression of CD133 and Nestin decreased (Figure 1B). Moreover, CD133+ GSCs formed tube-like structures *in vitro* when they were seeded onto Matrigel under hypoxia for 3 days (Figure 1C). Next, we established intracrainial glioma models with CD133+ GSCs containing the luciferase reporter gene (GSCs-luc), and 7.0T MRI scanning was regularly performed to observe tumor growth on T2W (Figure 1D). After 28 days, we detected human CD34 (hCD34) expression in tumors using immunohistochemistry (IHC) (Figure 1E). IF-double staining showed the co-expression of hCD34 and luciferase in microvessels (Figure 1F). These results are supported by the results of previous studies [7, 8, 10], which suggested that CD133+ GSCs could not only transdifferentiate into ECs *in vitro* under hypoxia but could also form functional microvessels *in vivo*.

### Microarray analysis of gene profiles in GSCs with and without hypoxia

To identify key genes in the process of transdifferentiation, we performed gene expression profiling using the Affimetrix GeneChip Human Exon 1.0ST Array. GSCs were maintained under hypoxia for 3 days and were compared with the control GSCs under normoxia. Differential gene expression was further analyzed based on the Gene Ontology (GO) and Kyoto Encyclopedia of Genes and Genomes (KEGG) databases. Path-Net and Signal-Net revealed significant differences in the pathways and genes involved in hypoxia in induced GSCs versus control GSCs (Supplementary Figures 1–2). According to Signal-Net results and with a focus on angiogenesis, we selected 8 differentially expressed candidate genes: ITGA3, ANGPTL4, BTG1, P4HA1, CLN5, MCM3, MCM7 and POLE (Supplementary Figure 2). qPCR was performed to confirm the fold changes of gene expression observed in microarrays (Supplementary Figure 3) and indicated that the expression levels of P4HA1 were significantly higher under hypoxia. Higher expression levels of P4HA1 in induced-GSCs were confirmed using western blot (Supplementary Figure 3).

### P4HA1 is highly expressed in high-grade gliomas and correlates with Ki67 and CD34 levels in human glioma specimens

**Figure 2:**
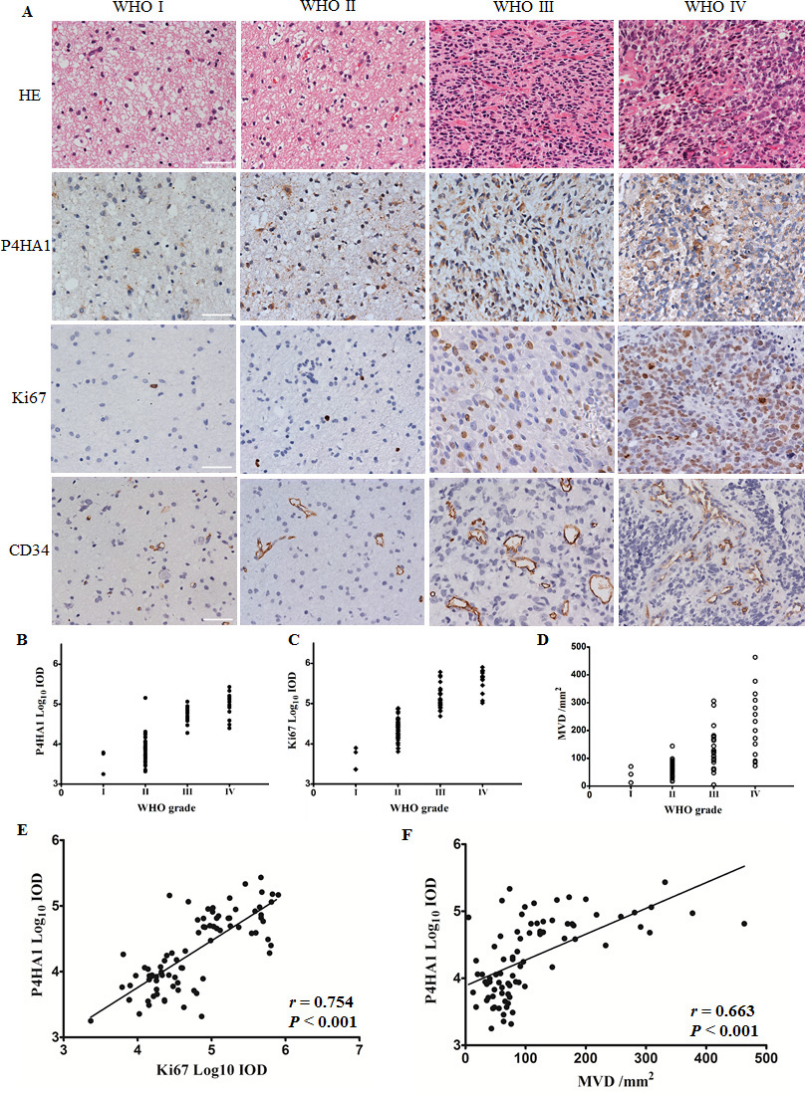
P4HA1 is overexpressed in high-grade gliomas and is correlated with Ki67 and MVD in human glioma specimens (**A**) HE and IHC staining of surgical specimens from four patients in four WHO grades. P4HA1 localized to the cytoplasm, and Ki67 was detected in the nucleus; CD34 was expressed in the membrane of endothelial cells that formed tube-like microvessels. Scale bar = 50 μm. (**B**) P4HA1 overexpression in high-grade gliomas. Significant differences were found in high-grade gliomas (WHO III and IV, *n* = 38, mean ± SD) compared to low-grade gliomas (WHO I and II, *n* = 43, mean ± SD) (*P* < 0.001) and between WHO III and IV grade gliomas using Student's *t* test (*P* < 0.05). (**C**–**D**) The levels of Ki67 and MVD increased as the WHO grade increased. (**E**) P4HA1 levels correlated with Ki67 levels in 81 human glioma specimens (*n* = 81, *r* = 0.754, *P* < 0.001). (**F**) A positive correlation between the levels of P4HA1 and MVD was also observed using the Spearman correlation test (*n* = 81, *r* = 0.663, *P* < 0.001).

In human glioma specimens, we determined the levels of the aforementioned candidate genes using IHC (Supplementary Table 1). P4HA1 was localized to the cytoplasm (Figure 2A) and was expressed in high-grade gliomas compared to low-grade gliomas, and a significant difference in P4HA1 expression was observed between WHO III and IV grade gliomas (Figure 2B). We then determined the levels of Ki67 and MVD (assessed by anti-human CD34 antibody) and found that both Ki67 and MVD levels increased as the grade increased (Figure 2C–2D). Positive correlations between the levels of P4HA1 and Ki67 (Figure 2E), as well as MVD (Figure 2F), were observed. These results are the first to reveal that P4HA1 was overexpressed in human gliomas. Correlation analyses indicated that P4HA1 may impact cell proliferation and microvessel formation. These findings were confirmed in the following *in vitro* study.

### Knockdown of P4HA1 suppresses GSC proliferation, tube formation and migration *in vitro*

**Figure 3:**
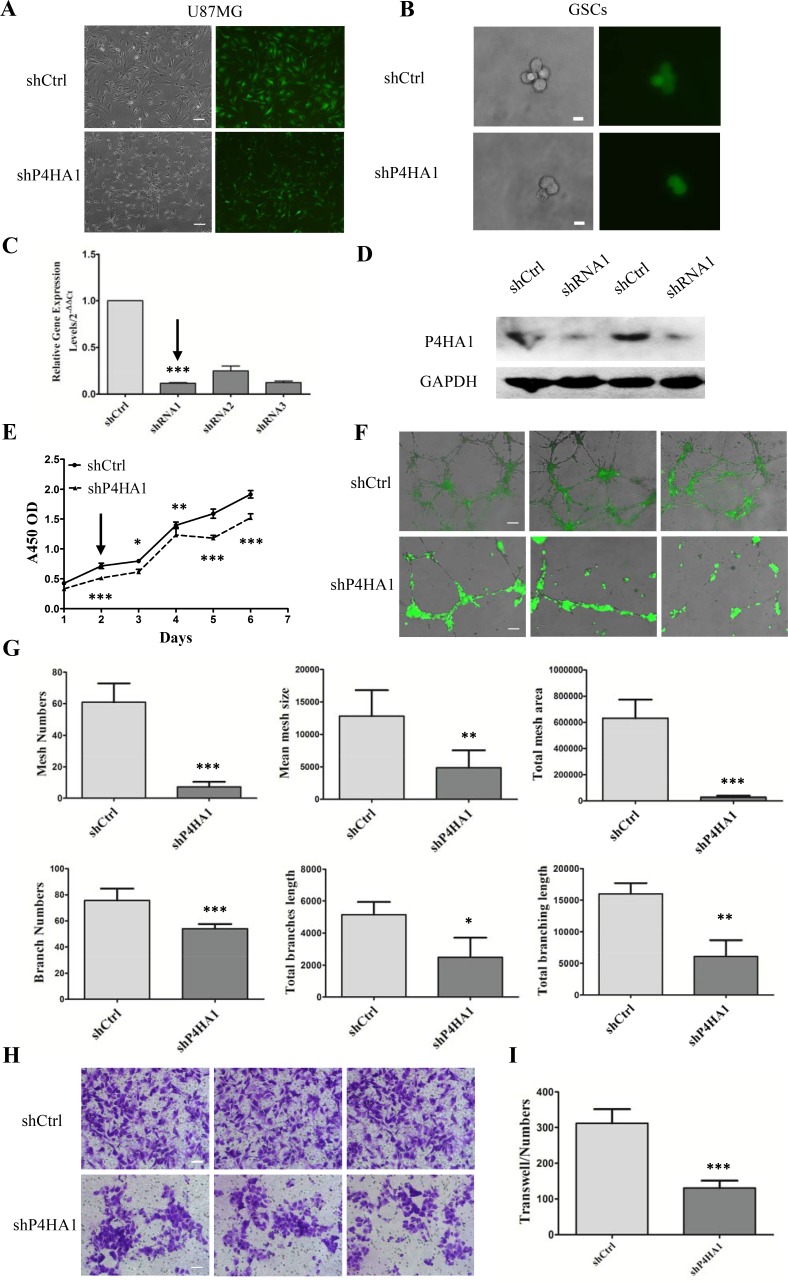
Knockdown of P4HA1 inhibits GSCs proliferation, tube formation and migration *in vitro* (**A**) U87MG cells were infected with lentiviral shRNA vectors labeled with GFP. Cells in the shP4HA1 group exhibited changes in morphology in contrast to shCtrl cells. Scale bar = 200 μm. (**B**) Single CD133+ cells from the two groups were placed in a 96-well plate. The growth of shP4HA1 cells was inhibited. Scale bar = 20 μm. (**C**–**D**) Quantitative PCR and western blotting analysis verified the effectiveness of shRNA1 transfection. (**E**) Proliferation of shP4HA1 and shCtrl GSCs under hypoxic conditions was determined from 1 to 6 days; the rate of shP4HA1 cell proliferation was significantly decreased from day 2. (**F**) A total of 1.5×10^5^ shP4HA1 and shCtrl GSCs were seeded onto Matrigel in 24-well plates and were incubated under hypoxic conditions for 3 days, during which time the cells formed tube-like structures. Bright-field images were merged with GFP fluorescent images. Scale bar = 200 μm. (**G**) Mesh numbers, mean mesh sizes, total mesh areas, branch numbers, total branch length and total branching length of the two groups were analyzed using ImageJ software. Significant differences were found between the shP4HA1 and shCtrl groups using Student's *t* test. (**H**) 5 × 10^4^ CD133+ cells were seeded onto Transwell membranes, and migrated cells were stained with cresyl violet and were counted using IPP software. Scale bar = 100 μm. (**I**) The migration ability of shP4HA1 GSCs was significantly attenuated compared to that of shCtrl GSCs (*P < 0.05; **P < 0.01; ***P < 0.001, mean ± SD).

U87MG cells were infected with three lentiviral shRNA vectors targeting P4HA1 (shP4HA1) and a control shRNA (shCtrl) (Figure 3A). Three days after infection, using FACS, GFP+ shP4HA1 and shCtrl cells were collected and cultured in serum-free medium for further analysis. After starvation in serum-free medium for more than one week, shP4HA1 and shCtrl cells were dispersed using Accutase and CD133+ cells were sorted using FACS. The growth rate of the single CD133+ cell in the shP4HA1 group was slower than the growth rate of cells in shCtrl group (Figure 3B). We performed qPCR to confirm that the mRNA level of P4HA1 had been downregulated, and we selected the most effective target-shRNA1 (Figure 3C). Western blotting confirmed the effectiveness of shRNA1 (Figure 3D).

We then determined the proliferation of shP4HA1 GSCs under hypoxia for 1 to 6 days. Compared to shCtrl GSCs, the proliferation rate of shP4HA1 GSCs was significantly lower on day 2 (Figure 3E). P4HA1 might be involved in GSC proliferation in response to hypoxia. To explore the effects of P4HA1 on tube formation, we seeded shP4HA1 GSCs or shCtrl GSCs onto Matrigel under hypoxia for 3 days (Figure 3F). Our results demonstrated that shCtrl GSCs could form tube-like structures with larger mean mesh sizes and a larger total mesh area, more mesh numbers and more branch numbers. In addition, a longer total branch length and a longer total branching length were observed in shCtrl group compared to shP4HA1 group (Figure 3G). These results suggested that knockdown of P4HA1 inhibits the ability of GSCs to form tube-like structures *in vitro*.

Furthermore, we determined the migration ability of shP4HA1 GSCs using Transwell chambers; cell migration is closely related to neovascularization. Our results showed that the migration ability of shP4HA1 GSCs was significantly inhibited compared to that of shCtrl GSCs (Figure 3H–3I). Together, these findings suggest that P4HA1 can regulate the proliferation and migration of GSCs and is involved in neovascularization.

### Knockdown of P4HA1 inhibits tumor growth and prolongs the overall survival time in xenograft models

**Figure 4:**
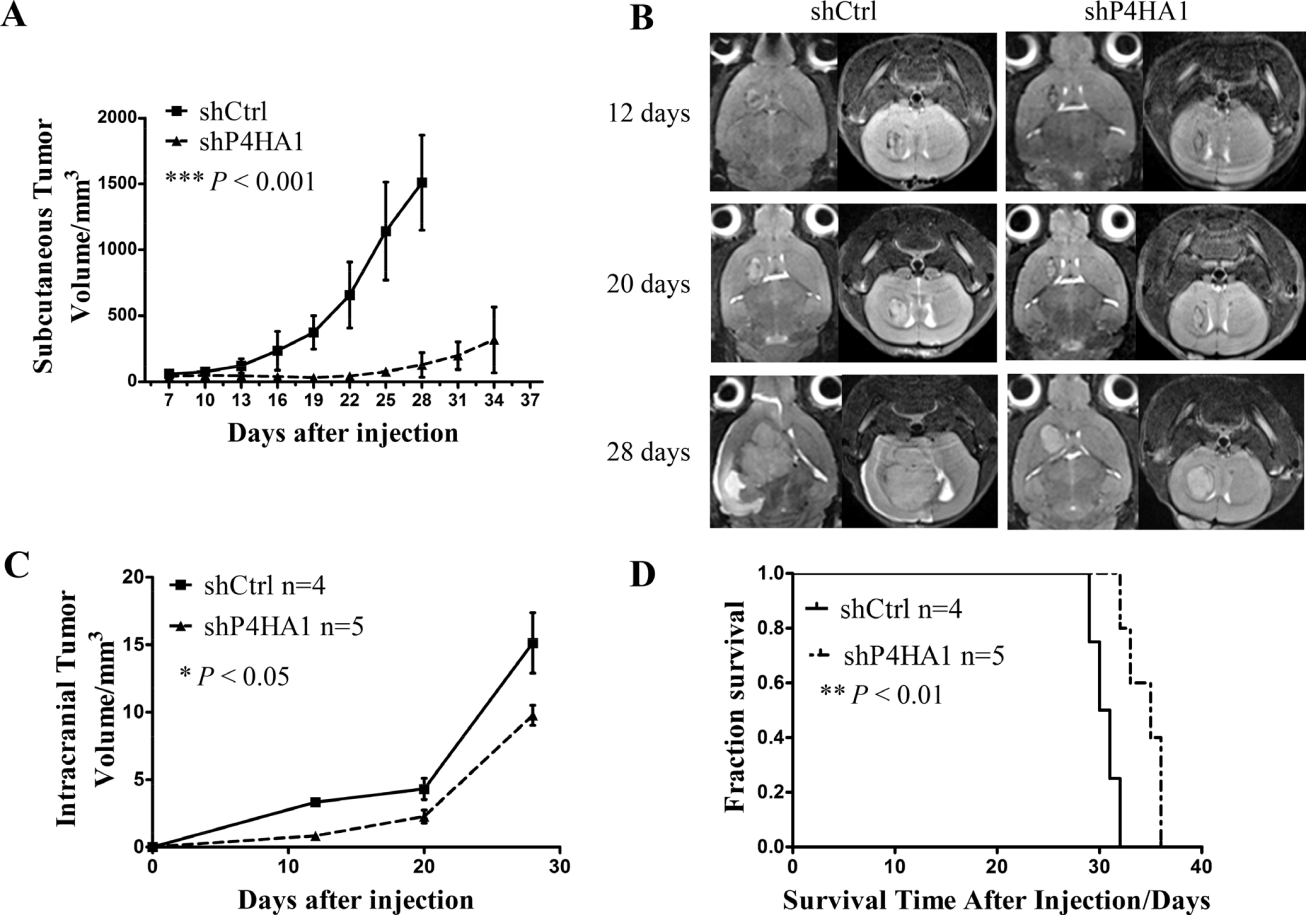
Knockdown of P4HA1 inhibits tumor growth and prolongs the OS of xenograft mice (**A**) The subcutaneous tumor growth rate was significantly lower in the shP4HA1 group compared to the shCtrl group. (**B**) Intracranial tumors were scanned using 7.0T MRI on days 12, 20 and 28. (**C**) Intracranial tumor volumes were measured using OsiriX software. Tumor volumes in the shP4HA1 group were significantly lower than in the shCtrl group. (**D**) Mice in the shP4HA1 group exhibited prolonged OS in contrast to the shCtrl group; differences were detected via log-rank analysis (shP4HA1 group, *n* = 5; shCtrl group, *n* = 4. *P < 0.05; **P < 0.01; ***P < 0.001, mean ± SD).

To evaluate the role of P4HA1 *in vivo*, we established subcutaneous and intracranial xenograft models using shP4HA1 GSCs and shCtrl GSCs. The subcutaneous tumor growth was measured every 3 days with vernier calipers, and it showed significant difference between the shP4HA1 group and the shCtrl group (Figure 4A). Intracranial tumor volumes were determined by MRI on days 12, 20 and 28 (Figure 4B) and revealed that tumor growth in shP4HA1 group was significantly slower compared to shCtrl group (Figure 4C). The median survival time (MS) of animals in the shP4HA1 group was 35 ± 2.19 days, and in the shCtrl group, the MS was 30.00 ± 0.67 days. Log-rank analysis of the two groups revealed that overall survival time (OS) was prolonged in animals in shP4HA1 group (Figure 4D). These results were consistent with the *in vitro* experiments and suggested that knockdown of P4HA1 could inhibit glioma growth. HE staining of sections from the mouse brain revealed pathological characteristics of the two groups of tumors (Supplementary Figure 4).

### Knockdown of P4HA1 inhibits transdifferentiation of GSCs into ECs and upregulates the expression of VEGF_165_b

**Figure 5:**
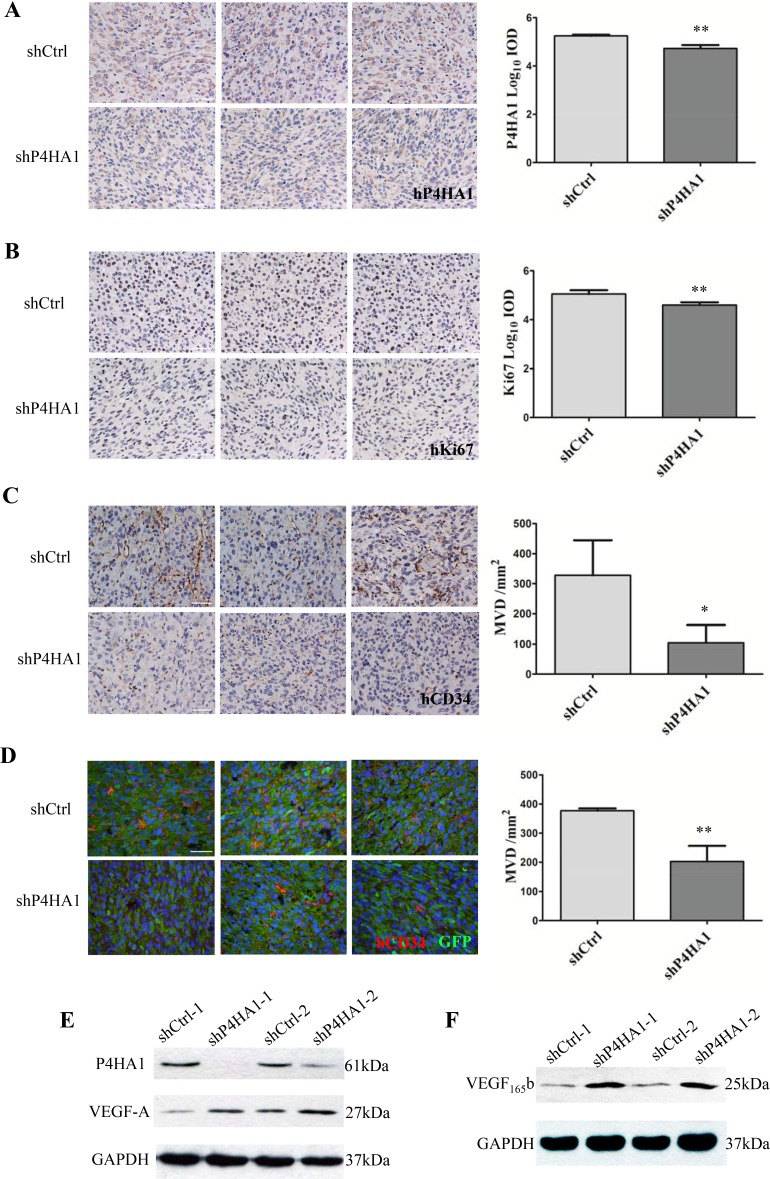
Knockdown of P4HA1 decreased the levels of Ki67 and MVD *in vivo* (**A**). IHC staining of P4HA1 demonstrated that the levels of P4HA1 in shP4HA1 tumors were significantly lower compared to those in shCtrl tumors. Scale bar = 50 μm.(**B**) The expression of Ki67 in shP4HA1 tumors was significantly lower than in shCtrl tumors. Scale bar = 50 μm. (**C**) The shP4HA1 and shCtrl tumor sections were determined using hCD34 antibody, and MVD of shP4HA1 tumors were significantly decreased. Scale bar = 50 μm. (**D**) The expression of MVD evaluated by IF was consistent with expression determined via IHC. Scale bar = 50 μm. (**E**) The expression levels of VEGF-A were upregulated in shP4HA1 GSCs. (**F**) The expression levels of VEGF_165_b were upregulated in sh P4HA1 GSCs. (*P < 0.05; **P < 0.01, mean ± SD).

To validate the role of P4HA1 in GSC proliferation *in vivo*, we determined the levels of Ki67 in tumor sections. Quantitative analysis of IHC results showed that the expression of P4HA1 was significantly lower in shP4HA1 tumors compared to shCtrl tumors (Figure 5A). IHC analysis also demonstrated that the expression of Ki67 was lower in shP4HA1 tumors (Figure 5B). To examine the effects of P4HA knockdown on GSCs transdifferentiation, we determined MVD in the tumor sections using the hCD34 antibody. Both of IHC and IF results revealed a significantly lower MVD in shP4HA1 tumors (Figure 5C–5D). Based on the above results, we tried to verify if the decrease of MVD was related to the expression of VEGF-A. Paradoxically, VEGF-A levels of shP4HA1 GSCs were upregulated in both of two independent experiments using western blotting (Figure 5E). Furthermore, we determined an anti-angiogenic alternative splicing-VEGF_165_b. Our results showed the levels of VEGF_165_b were upregulated in shP4HA1 GSCs (Figure 5E). These results indicated that P4HA1 may regulate the expression of VEGF_165_b in tumor neovascularization.

### Knockdown of P4HA1 decreases the levels of collagen IV and disrupts the structure of vascular BMs

**Figure 6:**
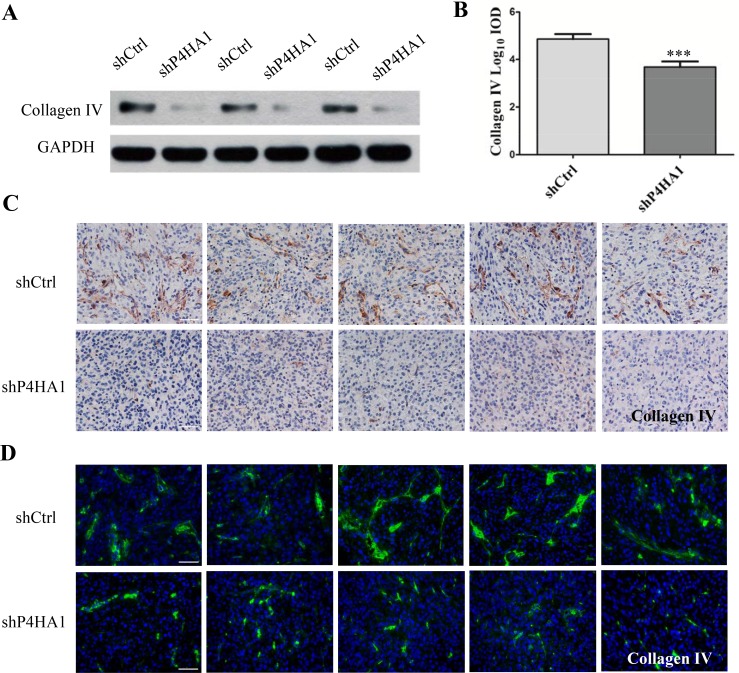
Knockdown of P4HA1 decreases the levels of collagen IV and disrupts the vascular basement membrane (**A**) The expression levels of collagen IV were significantly lower in shP4HA1 GSCs compared to shCtrl GSCs. (**B**) IHC staining revealed lower levels of collagen IV in the shP4HA1 group than in the shCtrl group. Scale bar = 50 μm. (**C**) In the shCtrl tumors, the structure of collagen IV appeared integral and continuous. Scale bar = 50 μm. (**D**) In the shP4HA1 tumors, the morphology of collagen IV was discontinuous, irregular and scattered. Scale bar = 50 μm (***P < 0.001, mean ± SD).

P4HA1, which is a critical isoform of the P4HA subunit, limits the activity of prolyl 4-hydroxylase (P4H) enzyme [16, 17]. P4H enzyme is responsible for biosynthesis of all types of collagens because it catalyzes the formation of hydroxyproline from proline residues [18, 19]. Evidence has shown that collagen IV is overexpressed in gliomas and localizes to the vascular BMs surrounding the ECs [20]. Therefore, we determined the levels of collagen IV in GSCs. Western blotting revealed that the level of collagen IV was lower in shP4HA1 GSCs (Figure 6A). Moreover, both IHC and IF staining revealed lower levels of collagen IV in the shP4HA1tumors (Figure 6B–6C). These results suggested that knockdown of P4HA1 could decrease the levels of collagen IV *in vitro* and *in vivo*. In addition, both IHC and IF staining of collagen IV in shCtrl tumors showed integral and continuous structures around the microvessels. In shP4HA1 tumors, the morphology of collagen IV fibers was discontinuous, irregular, and markedly different from that of control fibers (Figure 6C–6D). Because collagen IV is an essential components of the vascular BM, these results indicated that downregulation of P4HA1 could inhibit the biosynthesis of collagen IV and hence disrupt the structure of vascular BMs.

## DISCUSSION

The pattern of GSC-EC transdifferentiation was described by Ricci-Vitiani *et al*.[7] and Wang *et al*.[8], whose discovery may be involved in the resistence to the present anti-angiogenic therapy for GBMs, hence the key genes during this process could be new targets for anti-angiogenic therapy. Wang *et al*.[8] found that Notch1/2 and VEGFR1/2 were significantly upregulated in CD133+/CD144+ cells compared to CD133+/CD144- cells. Another study demonstrated that inhibition of Notch signaling weakens the self-renewal ability of GSCs, decreases the proportion of ECs in tumor tissues [21], but evidence for Notch inhibition directly blocking the transdifferentiation of GSCs into ECs is lacking. Therefore, the potential key genes involved in this process require further investigation.

Accumulating evidence reveals that hypoxia and HIFs play pivotal roles in tumor neovascularization. For example, VEGF-mediated angiogenesis can be activated by HIF-1α expression in GBM [15, 22]. Zhao *et al*.[10] reported that GSCs could express EC markers after being maintained under hypoxia for 4 hours *in vitro*. In addition, hypoxia and HIF-1α were reported to enhance the transdifferentiation of GBM cells into ECs [9, 23].

In the current study, we observed hCD34+ ECs that were transdifferentiated from CD133+ GSCs under hypoxia as well as functional microvessels expressing hCD34 and Luciferase in the brain tumors of xenograft mice. Microarray analysis showed the expression of different genes in normal GSCs versus hypoxia-induced GSCs. ITGA3, ANGPTL4, BTG1, P4HA1, CLN5, MCM3, MCM7 and POLE were ruled out as candidate genes. In an analysis of 81 human glioma specimens using IHC, the P4HA1 gene stood out: the level of P4HA1 were significantly higher in high-grade gliomas, it correlates with MVD and Ki67. These results indicated that P4HA1 implicates in proliferation and neovascularization in gliomas. Another study also detected a significant fold change in P4HA1 expression under hypoxia in GL261 cell lines [24]. Therefore, we focused on the function of P4HA1 and its role in cancer.

P4HA1 is a subunit of the rate-limiting enzyme P4H. P4H plays crucial roles in procollagen hydroxylation and secretion, and is essential for synthesizing all types of collagens [25]. In many hypoxia-related microarray analyses [24, 26, 27], P4HA1 had been identified as exhibiting significant changes in response to hypoxia. By inducing P4HA1 and P4HA2 expression in fibroblasts, HIF-1 enhences extracelluar matrix remodeling under hypoxia [28]. However, few studies have focused on the function of P4HA1 in cancers until recently. In human breast cancers, P4HA1 is overexpressed and correlated with patient prognosis [29]. Silencing HIF-1α blocks the expression of P4HA *in vitro*, and knockdown of P4HA1 inhibits tumor growth and metastasis in breast cancer *in vivo*. Additionally, overexpression of P4HA1 promotes tumor invasion and metastasis via the P4HA1-MMP1 pathway in prostate cancer [30]. In human gliomas, we first demonstrated that downregulation of P4HA1 could significantly suppress the proliferation and migration of GSCs during hypoxia *in vitro*. Meanwhile, an *in vivo* study demonstrated a significant inhibition of tumor growth and prolonged OS of xenograft mice due to downregulation of P4HA1. These results are consistent with the aforementioned studies in breast and prostate cancers.

To date, few studies have reported the effects of P4HA1 on neovascularization. Our studies revealed that knockdown of P4HA1 attenuates the ability of GSCs to form tube-like structures under hypoxia. We also showed knockdown of P4HA1 upregulates the expression of VEGF_165_b, which is an anti-angiogenic factor. Studies have demonstrated that VEGF_165_b inhibits tube formation of ECs *in vitro*, and prevents angiogenesis in mammary tissues [31–33]. In brain tumors of xenograft mice, the proportion of hCD34+ cells and MVD were significantly lower in shP4HA1 tumors. These results indicate that P4HA1 participates in the process of GSC-EC transdifferentiation in response to hypoxia, and it may regulate the expression of VEGF_165_b during tumor neovascularization. However, the mechanism on how P4HA1 regulating VEGF_165_b needs further investigation.

Holster *et al*.[34], who generated null mice with an inactivation of P4HA1 gene, reported that silencing of P4HA1 gene led to an absence of collagen IV in BMs and was associated with rupture of BM structures. Our results revealed that the levels of collagen IV in shP4HA1 GSCs were significantly lower than in shCtrl GSCs using western blotting. Similarly, IHC and IF staining of mouse brain tumors indicated that the levels of collagen IV in shP4HA1 tumors were significantly decreased, and the structures of collagen IV in shP4HA1 tumors appeared scattered and irregular compared to the continuous morphology of collagen IV observed in shCtrl tumors. These results indicated that downregulation of P4HA1 could not only decrease the synthesis of collagen IV but could also disrupt the structure of collagen IV, and hence compromise the integrity of vascular BMs in glioma tissue. Additionally, previous studies demonstrated that collagen IV, which localizes in the tumor vascular BMs, is upregulated in gliomas, and collagen IV can regulate angiogenesis via effects on structural integrity and assembly [20, 35]. Vascular BMs have been reported to contribute to the initiation of angiogenesis and the induction of ECs to form capillary-like structures [35, 36]. Taking all of these findings into account, we may conclude that knockdown of P4HA1 disrupts the integrity of tumor vessels via suppression of the collagen IV formation in glioma.

In summary, the present study is the first to elucidate the role of P4HA1 on proliferation, migration and tube formation in glioma cells. Downregulation of P4HA1 upregualtes the expression of VEGF_165_b, suppresses intracranial tumor growth and blocks the transdifferentiation of GSCs into ECs, inhibiting tumor neovascularization. P4HA1 also had an effect on collagen IV synthesis and the structural integrity of vascular BMs in glioma. Therefore, our study reveals a potential new target for anti-angiogenic treatment of glioma.

## MATERIALS AND METHODS

### Cell culture

Cells from the U87MG cell line (Cell Bank of Peking Union Medical College) were cultured in DMEM (Invitrogen) with 10% fetal bovine serum (FBS, Invitrogen). GSCs, which were sorted from U87MG cells using a CD133 antibody (Miltenyi) and flow cytometry (Beckman Coulter), were maintained in neurobasal medium with 20 ng/ml human EGF (Invitrogen), 20 ng/ml human bFGF (Invitrogen), 2% B27 (Invitrogen), and 2% glutamate (Invitrogen). In the differentiation-induction assay, GSCs were cultured under hypoxia with 1% O_2_ using an N_2_-O_2_ incubator (Thermo). U87-Luci (PerkinElmer), which expresses the firefly luciferase reporter gene, was cultured in 10% FBS medium with 1 μg/ml puromycin.

### Microarray analysis

GSCs were cultured under hypoxia for 3 days; control GSCs were cultured under normoxic conditions. Total RNA from the two group GSCs was extracted using TRIzol (Invitrogen) following the manufacturer's instructions. The cRNA preparation and microarray hybridization were performed as described before [24]. The Affimetrix GeneChip Human Exon 1.0ST Array was used to uncover the differential expression of genes via Gminix Shanghai Ltd. Analysis of differentially expressed genes and Signal-Net analysis were performed as previously described [37–39]. The expression levels of candidate genes in the induced-GSCs and control-GSCs were detected by qPCR.

### Lentivirus infection

The lentiviral vectors were designed and synthesized by Genechem Shanghai Ltd. The target sequences of shRNAs were provided in Supplementary Materials. The infection process was performed according to the manufacturer's instructions. U87MG cells were infected with a multiplicity of infection (MOI) of 2 and incubated for 6 hours. Three days after infection, cells were cultured in 10% FBS medium with 1 μg/ml puromycin (Sigma-Aldrich). Details on qPCR, Western blotting, flow cytometry and cell sorting can be found in Supplementary Materials.

### Cell proliferation, migration and tube formation assays

Cell proliferation was determined with a cell counting kit-8 assay (Dojindo). Migration assays were performed using Transwell plates (Corning). The reduced growth factor basement membrane matrix (Invitrogen) was placed in 24-well plates to determine tube formation abilities of GSCs. Details on these assays are provided in Supplementary Materials.

### Human glioma specimens

Eighty-one human glioma specimens were obtained from the Department of Neurosurgery, Beijing Tiantan Hospital affiliated to Capital Medical University, during 2013–2015 (Supplementary Figure 1). The histological diagnoses were confirmed by at least three experienced pathologists according to the WHO classification system [40]. Tissue collection was approved by the institutional review board of Beijing Tiantan Hospital affiliated to Capital Medical University, and informed consents were obtained prospectively. Detailed information for immunohistochemistry and assessment are provided in Supplementary Materials.

### Animal studies

Animal experiments were approved by Experimental Animal Ethics Committee of Beijing Neurosurgical Institute and were carried out in accordance with the NIH Guide for the Care and Use of Laboratory Animals. Details on establishment of intracranial and subcutaneous GSC tumor models are provided in Supplementary Materials.

### Statistical analysis

Statistical analysis was performed using SPSS 16.0 software. Student's *t* test was used to analyze the significance of two group results. Spearman correlation tests were used to determine the relationship among Ki67, CD34 and P4HA1 according to IHC staining results. All values are expressed as the mean ± SD. The overall survival time was compared using Log-rank method. *P* < 0.05 was considered statistically significant.

## SUPPLEMENTARY MATERIALS FIGURES AND TABLES




