# Supplementary material for: Knockdown of P4HA1 inhibits neovascularization via targeting glioma stem cell-endothelial cell transdifferentiation and disrupting vascular basement membrane
Source: Oncotarget. 2017 Mar 16;8(22):35877–89. doi: 10.18632/oncotarget.16270 (PMC5482624; doi:10.18632/oncotarget.16270)
Supplement: Supplementary file 1 [file oncotarget-08-35877-s001.pdf]

Knockdown of P4HA1 inhibits neovascularization via targeting glioma stem cell-endothelial cell transdifferentiation and disrupting vascular basement membrane

Supplementary Materials

Detailed information for materials and reagements

DMEM (Invitrogen, Carlsbad, CA, USA), fetal bovine serum (FBS, Invitrogen), CD133 antibody (AC133, Miltenyi, Bergisch Gladbach, Germany), flow cytometry (Beckman Coulter, Brea, CA, USA), N_2-O_2 incubator (Thermo, Waltham, MA, USA), U87-Luci (PerkinElmer, Waltham, MA, USA), puromycin (Sigma-Aldrich, St. Louis, MO, USA).

Target sequences of shRNAs

5′-AGTGCTAGTTGGCAACAAA-3′ (shRNA1), 5′-GAGATTTCTACCATAGAT-A-3′ (shRNA2), 5′- TC GTATTATTCGCTTCCAT-3′ (shRNA3), 5′-TTCTCCGA ACG-TGTCACGT-3′ (shCtrl).

Quantitative reverse-transcriptase-polymerase chain reaction (qPCR)

The total RNA extraction and qPCR procedures were performed as previously described.[1] The $2^{-\Delta\Delta Ct}$ method was used to analyze relative gene expression levels. The primer sequences were as follows: P4HA1, FW 5′-CAGAAGTACGAAATGCTG-TGCCG-3′, RV 5′-G CTTGTCCCATTCATCCTCCTGTT-3′, product length 145 bp. GAPDH, FW 5′- CTGGGCTACACTGAGCACC-3′, RV 5′-AAGTGGTCGTTGA-GGGCAATG-3′, product length 101 bp.

Western blotting

Protein extraction and western blotting were performed as previously described.[1] The primary antibodies anti-P4HA1 (1:200, ab127564, Abcam, Cambridge, UK), anti-VEGF-A (1:300, ab183100, Abcam), anti-VEGF165b (1:200, MAB3045, R&D), anti-GAPDH (1:2000, ab181602, Abcam), anti-collagen IV (1:1000, ab6586, Abcam) were used to detect protein expression.

Flow cytometry and cell sorting

Flow cytometry was performed using a cell sorter to screen out CD133+ GSCs from the U87MG cell line. Anti-human CD133 antibody (Miltenyi) was used to stain and sort positive GSCs as previously described.[2] Mouse phycoerythrin-IgG1 (Miltenyi) was used as an isotype control.

Cell proliferation

Cell proliferation was determined with a cell counting kit-8 assay (Dojindo).GSCs were initially pre-treated with Accutase and then seeded into 96-well plates at a density of 3000 cells per well in 100 µl medium. The cells were cultured for 1-7 days under hypoxic conditions, 10 µl of CCK8 solution was added to each well of the plate for 1 hour, and the absorbance at 450 nm was measured using a microplate reader. All data are presented as the mean ± SD from three experiments.

Cell migration assay

A total of 5×10^4 GSCs were seeded onto the upper compartment of a Transwell plate (24-well, 8 µm pore size, Corning) in 200 µl of serum-free DMEM, and the lower compartment was filled with 600 µl of 5% FBS medium. After incubation in the hypoxic N_2-O_2 incubator for 8 hours, cells on the upper surface of the membrane were undetectable, and the migrated cells on the lower surface were stained with 1% cresyl violet. The colored cells were counted, and all data represented the mean ± SD from three experiments.

Tube formation assay

The reduced growth factor basement membrane matrix (Geltrex, A1413202, Invitrogen) was thawed overnight at 4°C, and 50 µl/cm²/well of Geltrex was placed in 24-well plates without dilution. After incubating at 37°C for 30 minutes, 1.5×10^5 GSCs/well diluted in 1 ml

of 10% FBS medium were seeded on the solidified gel. All plates were incubated in the hypoxic N_2-O_2 incubator for 3 days. Images were randomly captured using a microscope (Olympus, Tokyo, Japan) with 100× magnification. Mesh numbers, mean mesh size, total mesh area, branch numbers, total branching length and total branch length were measured per field with ImageJ software (NIH website).

Immunohistochemistry, immunofluorescence and assessment

Immunohistochemical staining of human glioma samples, animal brain and subcutaneous tumors was performed as described before.[1] P4HA1, Ki67, hCD34 and collagen IV were detected by the following antibodies: anti-human P4HA1 (1:500, ab127564, Abcam), anti-human Ki67 (1:400, ab16667, Abcam), anti-human CD34 (1:100, sc-7324, Santa Cruz, CA, USA, it does not react with mouse-originated vessels), and anti-collagen IV (1:500, ab6586, Abcam). Five images from each slice were randomly captured with a camera (Jenoptik, Jena, Germany) coupled to a microscope (Zeiss, Oberkochen, Germany) at 400× magnification. Expression levels of P4HA1, Ki67 and collagen IV were analyzed with Image Pro Plus software 5.0 (IPP) (Media Cybernetics). Density mean, area sum, and integrated optical density (IOD) were determined. The levels of microvessel density (MVD) were assessed by hCD34 antibody binding following the principles described previously.[3] Five fields (0.079 mm^2 per field) of each tumor section were counted in the area of highest vascular density at 400× magnification. All figures in the text report area as mm^2. Immunofluorescence was performed as previously described.[4] hCD34+ cells were labeled with Alexa Fluor 647 (1:2000, ab150115, Abcam), and EGFP or Luciferase-positive cells were detected by anti-EGFP (1:400, ab184601, Abcam) or anti-luciferase (1:400, ab21176, Abcam) antibodies tagged with FITC (1:2000, ab6785/ab6717, Abcam). Photos were captured with a microscope and confocal microscope (Leica, Wetzlar, Germany).

Establishment of intracranial and subcutaneous GSC tumor models

Four- to six-week-old BALB/c-nu mice were purchased from Beijing HFK Bioscience Ltd. Animal experiments were approved by Experimental Animal Ethics Committee of Beijing Neurosurgical Institute and were carried out in accordance with the NIH Guide for the Care and Use of Laboratory Animals. After being anesthetized with isoflurane, mice were stabilized in a stereotactic apparatus (KOPF940). A total of 5×10^5 GSCs were injected into the brain to establish intracranial tumor models as previously described.[5] To establish subcutaneous GSCs tumor models, 1×10^6 GSCs in 100 µl PBS were injected directly into the dorsal subcutaneous tissue close to the right forelimb. Subcutaneous tumor size was measured every 3 days with a vernier caliper, and tumor volume was calculated as follows: $V = \pi/6 \times \text{Length} \times \text{Width}^2$ (mm^3).

Magnetic resonance imaging (MRI)

Mice were continuously anesthetized with isoflurane and were examined using a 7.0T/30 cm small-animal MRI scanner (Bruker BioSpin, Billerica, MA, USA). T2-weight (T2W) images were acquired using a rapid acquisition with relaxation enhancement (RARE) pulse sequence (axial view: TR = 3380 ms, TE = 41 ms, matrix = 320×256, slice thickness = 0.5 mm; coronal view: TR = 4030 ms, TE = 50 ms, matrix = 320×384, slice thickness = 0.3 mm). The maximal anteroposterior diameter (L), transverse diameter (W) and height (H) were measured with OsiriX software. Tumor volume was calculated as follows: $V = \pi/6\times L\times W\times H$ (mm^3).

SUPPLEMENTARY REFERENCES

1. Zhou Y, Jin G, Mi R, Dong C, Zhang J, Liu F. The methylation status of the platelet-derived growth factor-B gene promoter and its regulation of cellular proliferation following folate treatment in human glioma cells. Brain Res. 2014; 1556:57–66.
2. Wang Z, Wang B, Shi Y, Xu C, Xiao HL, Ma LN, Xu SL, Yang L, Wang QL, Dang WQ, Cui W, Yu SC, Ping YF, et al. Oncogenic miR-20a and miR-106a enhance the invasiveness of human glioma stem cells by directly targeting TIMP-2. Oncogene. 2015; 34:1407–1419.
3. Bottini A, Berruti A, Bersiga A, Brizzi MP, Allevi G, Bolsi G, Aguggini S, Brunelli A, Betri E, Generali D, Scaratti L, Bertoli G, Alquati P, et al. Changes in microvessel density as assessed by CD34 antibodies after primary chemotherapy in human breast cancer. Clin Cancer Res. 2002; 8:1816–1821.
4. Macarthur KM, Kao GD, Chandrasekaran S, Alonso-Basanta M, Chapman C, Lustig RA, Wileyto EP, Hahn SM, Dorsey JF. Detection of brain tumor cells in the peripheral blood by a telomerase promoter-based assay. Cancer Res. 2014; 74:2152–2159.
5. Lal S, Lacroix M, Tofilon P, Fuller GN, Sawaya R, Lang FF. An implantable guide-screw system for brain tumor studies in small animals. J Neurosurg. 2000; 92:326–333.

Supplementary Table 1: Medical information of the 81 patients. See Supplementary_Table_1

Supplementary Table 2: Candidate genes

Candidate genes	Style	Gene chip fold change (ECs/GSCs)	qPCR fold change (ECs/GSCs)	Pathway
**P4HA1**	up	3.060002857	5.465297761	Arginine and proline metabolism
**POLE**	down	0.380757141	0.561119649	DNA replication
**MCM3**	down	0.382878883	0.378631944	DNA replication
**MCM7**	down	0.355867231	0.417522223	DNA replication
**ITGA3**	down	0.466070143	0.520077082	Hematopoietic cell lineage
**CLN5**	up	3.157826702	3.527873653	Lysosome
**ANGPTL4**	up	2.099375159	3.971771134	Angiogenesis
**BTG1**	up	2.019737777	3.282186	Angiogenesis

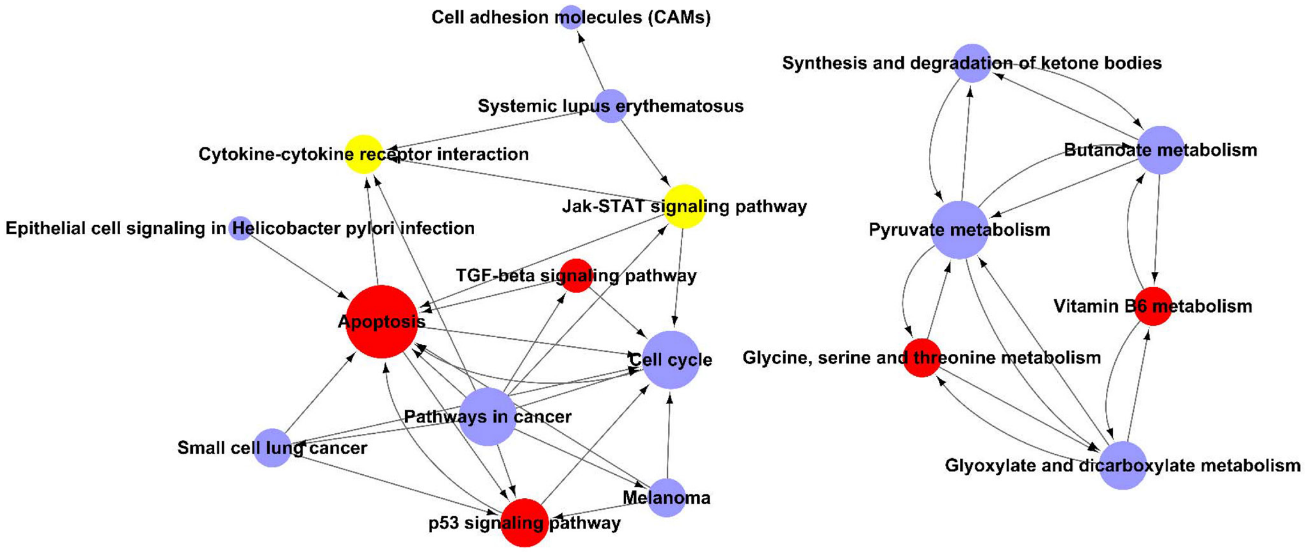

Supplementary Figure 1: Path-Net analysis of significantly differential genes. Red circles: upregulated genes related pathways. Blue circles: downregulated genes related pathways. Yellow circles: Both of up- and downregulated genes related pathways.

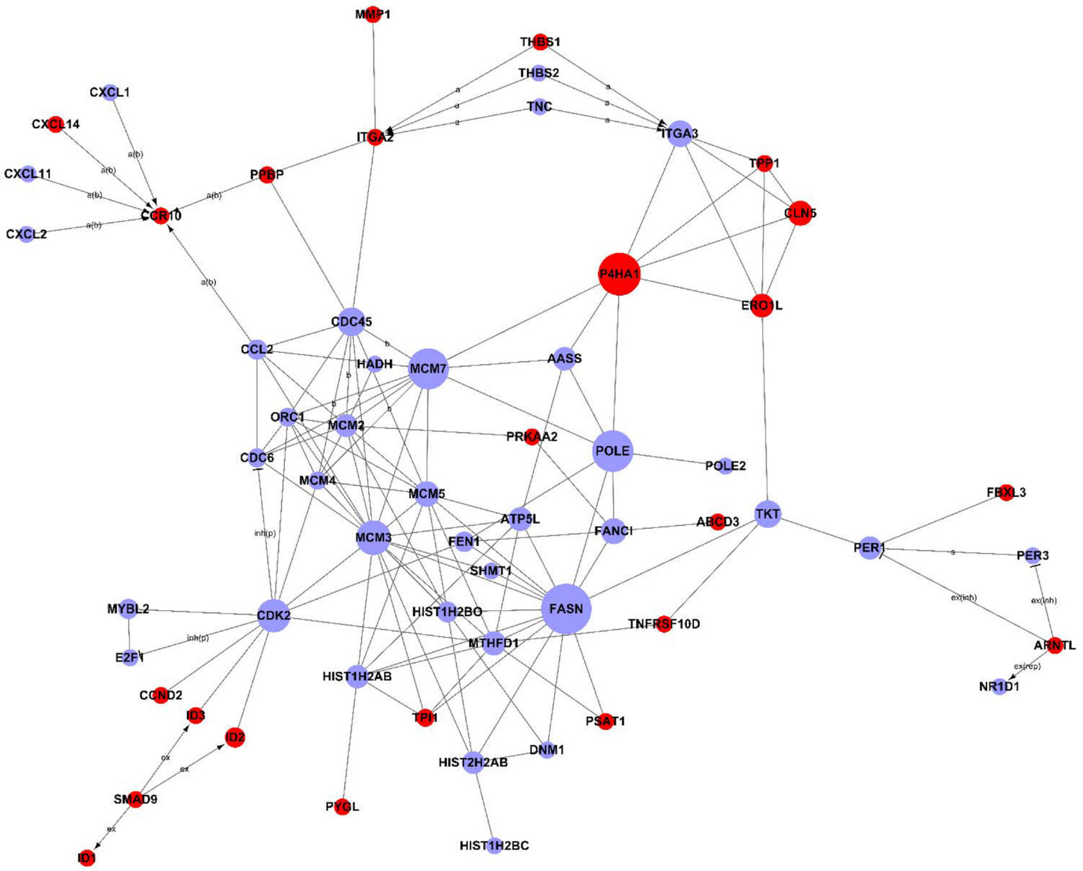

Supplementary Figure 2: Signal-Net analysis of significantly differential genes. Red circles: upregulated genes; Blue circles: downregulated genes; (**A**) activation; a(b): activation(binding/association); (**B**) binding/association; ex: gene expression; ex(inh): expression(inhibition); ex(rep): expression(repression); s: state change.

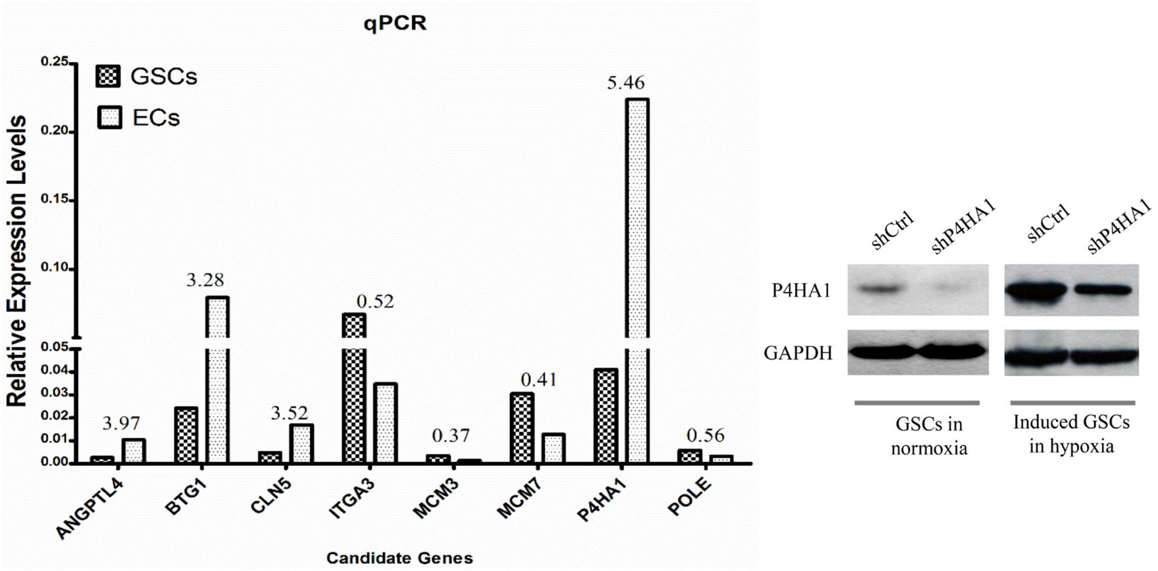

Supplementary Figure 3: mRNA fold changes of candidate genes in the induced GSCs (ECs) versus normal GSCs using qPCR. And higher expression levels of P4HA1 in hypoxia induced GSCs were confirmed using western blot.

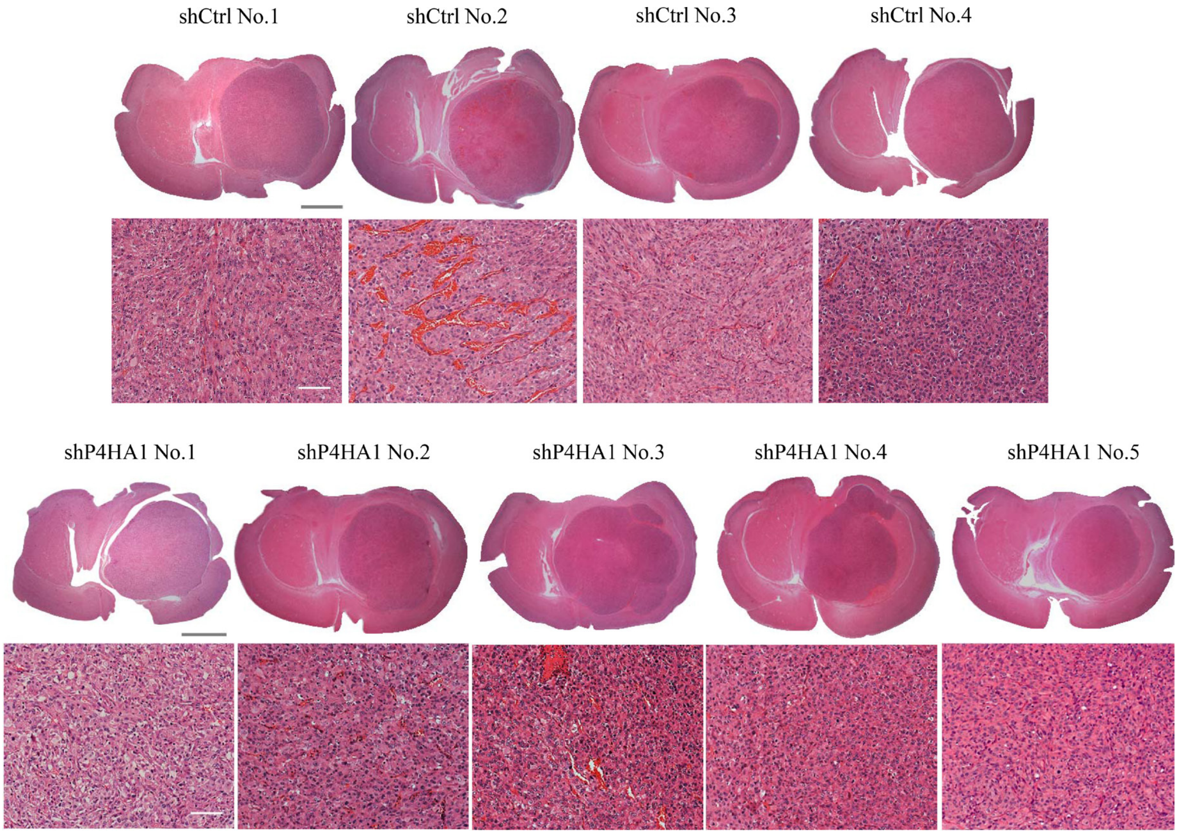

Supplementary Figure 4: HE staining of the two groups of mice brain tumors. Gray scale bar = 1 cm, White scale bar = 500 μm.
