# Supplementary material for: Knockdown of P4HA1 inhibits neovascularization via targeting glioma stem cell-endothelial cell transdifferentiation and disrupting vascular basement membrane
Source: Oncotarget. 2017 Mar 16;8(22):35877–89. doi: 10.18632/oncotarget.16270 (PMC5482624; doi:10.18632/oncotarget.16270)
Supplement: Supplementary file 2 [file oncotarget-08-35877-s002.docx]

| **Table S1 Medical information of the 81 patients** | | | | | | | |
| --- | --- | --- | --- | --- | --- | --- | --- |
| Patients  No. | Age years | Gender | Pathological  Diagnosis | WHO  grade | Ki-67  log_10_ IOD | MVD /mm^2^ | P4HA1  log_10_ IOD |
| 1 | 44 | M | GBM | IV | 5.650057 | 281.012658 | 4.980387989 |
| 2 | 25 | M | AOA | III | 5.070569 | 124.050633 | 4.656635528 |
| 3 | 42 | M | GBM | IV | 5.589105 | 258.227848 | 4.919859 |
| 4 | 43 | M | OA | II | 4.141571 | 45.569620 | 3.5488701 |
| 5 | 30 | M | FA | II | 3.806273 | 17.721519 | 4.261240479 |
| 6 | 42 | M | OA | II | 3.888649 | 17.721519 | 3.570517099 |
| 7 | 61 | M | OA | II | 4.506045 | 35.443038 | 3.911948976 |
| 8 | 40 | M | OA | II | 4.096119 | 20.253165 | 4.059810714 |
| 9 | 31 | M | OA | II | 4.480256 | 60.759494 | 3.774841863 |
| 10 | 49 | F | GBM | IV | 5.803124 | 86.075949 | 4.396826575 |
| 11 | 44 | M | OA | II | 4.431850 | 60.759494 | 5.158127087 |
| 12 | 49 | M | GBM | IV | 5.070240 | 463.291139 | 4.813654318 |
| 13 | 32 | F | GA | II | 4.763644 | 37.974684 | 3.711117132 |
| 14 | 32 | M | OA | II | 4.885451 | 73.417722 | 3.891606542 |
| 15 | 61 | F | OA | II | 4.543829 | 48.101266 | 3.828466268 |
| 16 | 28 | F | AA | III | 5.095303 | 129.113924 | 4.846182776 |
| 17 | 39 | M | OA | II | 4.866430 | 75.949367 | 3.317262386 |
| 18 | 56 | M | AA | III | 5.115978 | 58.227848 | 4.626910288 |
| 19 | 59 | M | AOA | III | 4.891177 | 177.215190 | 4.808951639 |
| 20 | 41 | M | OA | II | 4.645234 | 78.481013 | 4.3122337 |
| 21 | 54 | F | AOA | III | 4.824923 | 164.556962 | 4.592641334 |
| 22 | 40 | M | AA | III | 4.686646 | 98.734177 | 5.063734497 |
| 23 | 41 | M | PA | I | 3.898523 | 12.658228 | 3.787543364 |
| 24 | 45 | M | OD | II | 4.263292 | 65.822785 | 3.654611827 |
| 25 | 66 | M | PA | I | 3.791222 | 70.886076 | 3.763227703 |
| 26 | 61 | F | GBM | IV | 5.823113 | 200.000000 | 5.177748064 |
| 27 | 14 | M | OA | II | 4.591050 | 27.848101 | 4.056250809 |
| 28 | 40 | M | PPA | II | 4.548962 | 73.417722 | 3.71724507 |
| 29 | 42 | M | OA | II | 4.464256 | 78.481013 | 4.282020908 |
| 30 | 47 | M | AA | III | 4.952702 | 93.670886 | 4.953250773 |
| 31 | 62 | M | GBM | IV | 5.763272 | 232.911392 | 4.488879748 |
| 32 | 56 | F | GBM | IV | 5.598831 | 91.139241 | 4.589932399 |
| 33 | 44 | F | PA | I | 3.366740 | 43.037975 | 3.249273474 |
| 34 | 26 | F | FA | II | 4.422868 | 83.544304 | 3.946222871 |
| 35 | 40 | F | OD | II | 4.195051 | 32.911392 | 3.945629856 |
| 36 | 48 | M | FA | II | 4.364053 | 48.101266 | 3.566099003 |
| 37 | 71 | M | AOA | III | 5.243290 | 108.860759 | 4.812522189 |
| 38 | 44 | F | AA | III | 4.893137 | 306.329114 | 4.679995094 |
| 39 | 42 | M | GA | II | 4.626158 | 63.291139 | 3.455615802 |
| 40 | 33 | M | OA | II | 4.606200 | 50.632911 | 4.054183038 |
| 41 | 52 | F | AOA | III | 4.911299 | 124.050633 | 4.674817474 |
| 42 | 39 | M | AOD | III | 4.811908 | 179.746835 | 4.786245204 |
| 43 | 26 | M | AOA | III | 5.028867 | 129.113924 | 4.685542157 |
| 44 | 52 | M | OD | II | 4.798166 | 35.443038 | 3.669172917 |
| 45 | 52 | F | AOA | III | 4.977835 | 124.050633 | 4.69895422 |
| 46 | 31 | F | FA | II | 4.153698 | 78.481013 | 3.488544659 |
| 47 | 36 | M | GBM | IV | 5.667829 | 331.645570 | 5.432894298 |
| 48 | 40 | M | AOA | III | 5.226791 | 169.620253 | 4.81240821 |
| 49 | 41 | F | GBM | IV | 5.902108 | 151.898734 | 5.166214759 |
| 50 | 30 | F | AOA | III | 5.541587 | 182.278481 | 4.583233643 |
| 51 | 35 | M | AA | III | 5.325716 | 217.721519 | 4.946734627 |
| 52 | 50 | M | OA | II | 4.253745 | 70.886076 | 3.92051641 |
| 53 | 60 | F | AOA | III | 5.669742 | 144.303797 | 4.862436837 |
| 54 | 61 | M | GBM | IV | 5.809437 | 308.860759 | 5.05890188 |
| 55 | 58 | F | GBM | IV | 5.456153 | 73.417722 | 5.334102394 |
| 56 | 26 | M | AA | III | 5.014784 | 5.063291 | 4.90803168 |
| 57 | 40 | F | AOA | III | 4.987712 | 48.101266 | 4.472998015 |
| 58 | 13 | M | AA | III | 5.254783 | 86.075949 | 4.69303137 |
| 59 | 48 | F | OA | II | 4.332547 | 40.506329 | 3.945509637 |
| 60 | 51 | M | OD | II | 4.321459 | 40.506329 | 3.992859167 |
| 61 | 46 | M | OA | II | 3.975632 | 88.607595 | 3.937744336 |
| 62 | 48 | M | AA | III | 5.669711 | 118.987342 | 4.818168588 |
| 63 | 49 | M | FA | II | 3.885144 | 68.354430 | 3.567065294 |
| 64 | 23 | M | FA | II | 4.256647 | 45.569620 | 3.728408195 |
| 65 | 41 | M | OA | II | 4.156687 | 98.734177 | 3.87715162 |
| 66 | 42 | F | OD | II | 4.365483 | 55.696203 | 3.551166174 |
| 67 | 37 | F | FA | II | 4.311156 | 58.227848 | 4.070582086 |
| 68 | 45 | F | AOA | III | 5.698412 | 291.139241 | 4.765341401 |
| 69 | 36 | F | OA | II | 4.023647 | 63.291139 | 3.356113714 |
| 70 | 65 | M | GBM | IV | 5.017748 | 377.215190 | 4.969999263 |
| 71 | 52 | F | GBM | IV | 5.246043 | 113.924051 | 5.117934684 |
| 72 | 53 | M | GBM | IV | 5.679863 | 172.151899 | 5.208537835 |
| 73 | 57 | M | OA | II | 4.394856 | 96.202532 | 4.244773838 |
| 74 | 50 | M | FA | II | 4.526478 | 91.139241 | 4.178180113 |
| 75 | 48 | F | OA | II | 4.215584 | 70.886076 | 3.623602079 |
| 76 | 36 | M | AOA | III | 5.366841 | 106.329114 | 4.674065232 |
| 77 | 56 | M | AOA | III | 5.784113 | 63.291139 | 4.281839401 |
| 78 | 27 | M | OD | II | 4.141488 | 78.481013 | 4.037644937 |
| 79 | 53 | F | OA | II | 4.158726 | 55.696203 | 3.934732724 |
| 80 | 47 | F | FA | II | 4.365484 | 144.303797 | 4.166136082 |
| 81 | 35 | F | OA | II | 4.254156 | 60.759494 | 3.862046062 |
| OA, oligoastrocytoma; OD, oligodendroglioma; GBM, glioblastoma; AA, anaplastic astrocytoma; AOA, anaplastic oligoastrocytoma; AOD, anaplastic oligodendroglioma; PPA, protoplasmic astrocytoma; PA, pilocytic astrocytoma; FA, fibrillary astrocytoma; GA, gemistocytic astrocytoma | | | | | | | |
